# Synthesis of MeOH and DME From CO_2_ Hydrogenation Over Commercial and Modified Catalysts

**DOI:** 10.3389/fchem.2022.903053

**Published:** 2022-06-03

**Authors:** Rafaelle G. Santiago, Juliana A. Coelho, Sebastião M. P. de Lucena, Ana Paula S. Musse, Marcio de F. Portilho, Enrique Rodriguez-Castellón, Diana C. S. de Azevedo, Moises Bastos-Neto

**Affiliations:** ^1^ Grupo de Pesquisa em Separações por Adsorção (GPSA), Department of Chemical Engineering, Federal University of Ceará, Fortaleza, Brazil; ^2^ PETROBRAS/CENPES, Cidade Universitária, Rio de Janeiro, Brazil; ^3^ Department of Inorganic Chemistry, Facultad de Ciencias, Universidad de Málaga, Málaga, Spain

**Keywords:** CO_2_, methanol, DME, catalysis, fixed bed

## Abstract

Growing concern about climate change has been driving the search for solutions to mitigate greenhouse gas emissions. In this context, carbon capture and utilization (CCU) technologies have been proposed and developed as a way of giving CO_2_ a sustainable and economically viable destination. An interesting approach is the conversion of CO_2_ into valuable chemicals, such as methanol (MeOH) and dimethyl ether (DME), by means of catalytic hydrogenation on Cu-, Zn-, and Al-based catalysts. In this work, three catalysts were tested for the synthesis of MeOH and DME from CO_2_ using a single fixed-bed reactor. The first one was a commercial CuO/γ-Al_2_O_3_; the second one was CuO-ZnO/γ-Al_2_O_3_, obtained *via* incipient wetness impregnation of the first catalyst with an aqueous solution of zinc acetate; and the third one was a CZA catalyst obtained by the coprecipitation method. The samples were characterized by XRD, XRF, and N_2_ adsorption isotherms. The hydrogenation of CO_2_ was performed at 25 bar, 230°C, with a H_2_:CO_2_ ratio of 3 and space velocity of 1,200 ml (g cat · h)^−1^ in order to assess the potential of these catalysts in the conversion of CO_2_ to methanol and dimethyl ether. The catalyst activity was correlated to the adsorption isotherms of each reactant. The main results show that the highest CO_2_ conversion and the best yield of methanol are obtained with the CZACP catalyst, very likely due to its higher adsorption capacity of H_2_. In addition, although the presence of zinc oxide reduces the textural properties of the porous catalyst, CZAWI showed higher CO_2_ conversion than commercial catalyst CuO/γ-Al_2_O_3_.

## Introduction

Carbon dioxide (CO_2_) is emitted mainly from the combustion of fossil fuels in power generation. The increasing concentration of CO_2_ in the atmosphere is related to global warming and climate changes. Replacement of fossil fuels with renewable energy sources is likely to reduce this problem, but this is only a long-term solution since the current world energy matrix is highly dependent on coal and oil-fired power plants. Thus, in recent decades, technologies aiming at carbon capture and storage (CCS) have been developed as a potential short-term solution to decrease or stabilize CO_2_ concentration in the atmosphere. Concomitantly, this greenhouse gas has also been investigated as a potential carbon feedstock, and a growing number of studies have been addressing the conversion of CO_2_ into useful chemicals. It seems a more attractive alternative in comparison to geological storage, which has been the main post-capture destination for CO_2_.

The main challenge in converting CO_2_ to other chemicals is its relatively low reactivity so high reaction temperatures and/or pressures are usually required for its reaction. Nevertheless, advances in heterogeneous catalysis show promise to allow these reactions to occur under milder conditions. Different approaches drive the current research about CO_2_ utilization as a feedstock (Song, 2006). Industrially, CO_2_ is most commonly used to react with ammonia to obtain urea and with phenol to produce salicylic acid. Emerging applications of CO_2_ conversion involve methane (CH_4_) reforming (Chen et al., 2019; Chong et al., 2019) and tri-reforming (Damanabi et al., 2019), synthesis of dimethyl carbonate (DMC) (Li et al., 2017; Sun et al., 2020), and hydrogenation that can be directed to form various products depending on the catalyst and the reaction conditions used (Wang et al., 2011; Ye et al., 2019; Saeidi et al., 2021; Yang et al., 2007).

Among different applications, CO_2_ hydrogenation is a promising route to produce chemicals and fuels since the net reaction is usually exothermic, requiring less energy input (Li et al., 2018; Marlin et al., 2018). The key advantage of this type of catalytic conversion is its potential to transform large amounts of CO_2_ with acceptable kinetics and considerable process efficiency (Alvarez et al., 2017). The main products formed from the hydrogenation of CO_2_ are carbon monoxide, hydrocarbons, methanol, dimethyl ether (DME), higher alcohols, formic acid, and formamides (Wang et al., 2011).

Methanol is one of the main chemical commodities traded worldwide, with an expected demand of 190 Mt/year by 2030 (Battaglia et al., 2021). Its importance is mainly regarded from its value as an intermediate chemical, leading to products such as formaldehyde, DME, and acetic acid. It may also be used as a feedstock for the production of olefins, which are intermediates for the formation of several chemicals consumed in daily life, including paints, plastics, resins, adhesives, and antifreeze additives (Olah et al., 2008; Jadhav et al., 2014; Adnan and Kibria, 2020; Kim et al., 2021).

The “Methanol Economy” concept emerged from the fact that methanol may offer a viable solution for the efficient storage and transportation of sustainable energy, considering that it is produced by reacting CO_2_ captured from large emitters and H_2_ obtained from renewable sources. Taking into account the challenges of storing and transporting hydrogen in liquid or compressed forms, methanol emerges as a promising liquid carrier (Ganesh, 2014; Bowker, 2019).

Among the chemicals obtained from methanol, DME has the potential to be deployed as a fuel due to its physicochemical properties. Not only is it similar to liquefied petroleum gas (LPG) with low emissions of NOx, SOx, and particulates (Arcoumanis et al., 2008; Kim et al., 2008; Marchionna et al., 2008; Bonura et al., 2017; Ateka et al., 2018), but it is also an important chemical intermediate in the production of light olefins and gasoline (Li et al., 2019; Magomedova et al., 2019). DME production from CO_2_ occurs in two steps: first, methanol is obtained from CO_2_ hydrogenation and then it is dehydrated to DME (An et al., 2008; Ateka et al., 2017). The main challenge of the direct DME synthesis is to develop an efficient multifunctional catalyst that has metal sites for methanol synthesis and acid sites for methanol dehydration to DME, both sites being highly selective to avoid the formation of by-products.

Regarding the catalysts applied in the CO_2_ hydrogenation to methanol, copper is one of the most suitable metals due to its high activity at lower temperatures, low cost, diverse oxidation states, and high interactivity with other materials (Murthy et al., 2021). These features render copper-based catalysts probably with the best performance in converting CO_2_ to methanol as compared to other metals (Catizzone et al., 2018). Furthermore, the occurrence of zinc species in copper-based catalysts has been reported to promote methanol synthesis significantly (Chinchen et al., 1986; Nakamura et al., 1995; Ma et al., 1998; Liu et al., 2003; Ahouari et al., 2013). The presence of ZnO leads to higher dispersion of Cu, thus preventing the agglomeration of metal particles and maintaining adequate copper surface area in the catalyst. This improves the resistance of Cu particles to poisoning and enhances CO_2_ adsorption on the catalyst surface, providing higher availability of active sites to the reactants (Alvarez et al., 2017; Catizzone et al., 2018). Aluminum oxide combined with Cu–Zn–based catalysts has also been reported as a stable catalyst for methanol synthesis (Aguayo et al., 2007; Naik et al., 2011; Ereña et al., 2013). *γ*-Al_2_O_3_ has been extensively studied as a catalyst for the dehydration of methanol to DME due to its low cost, good thermal stability, and high specific surface area (Aboul-Fotouh, 2014). In addition to the nature of the active metal in the catalyst composition, the preparation methods also may affect the physicochemical and morphological properties, such as total surface area, metal dispersion, and crystallinity, which could influence the catalytic activity (Wambach et al., 1999). The degree of interfacial contact, for instance, is higher for materials prepared by coprecipitation than by impregnation, and it is a crucial factor for the catalytic performance (Koeppel et al., 1992). On the other hand, the presence of residual precipitant agents during the calcination of the precursors by the coprecipitation method tends to promote the agglomeration of copper particles, which decreases the metal dispersion and has a negative influence on the catalytic performance (Prieto et al., 2013; Alvarez et al., 2017).

The understanding of what occurs at the molecular level in these catalytic reactions is still under development, and there is no consensus about the mechanisms involved in the CO_2_ hydrogenation reactions to produce methanol and DME (Alvarez et al., 2017). Because adsorption is one of the steps in the conversion of reactants into products, the evaluation of catalysts from the perspective of the adsorption phenomenon can shed some light on these questions.

The main purpose of this work is to provide insights into the CO_2_ hydrogenation mechanism by correlating the catalytic activity with the adsorption of each reactant on the catalyst. Two catalysts were investigated: a commercial catalyst based on copper and the other one, which was the former loaded with zinc by wet impregnation. These materials were compared with those using a copper-zinc catalyst (CZA) obtained by coprecipitation since the latter is the most applied catalyst in the methanol production by carbon hydrogenation. Characterization techniques were carried out to identify the features that impact the catalytic activity of these materials in the methanol formation from CO_2_ hydrogenation.

## Materials and Methods

### Catalysts for CO_2_ Conversion

Commercial copper (II) oxide on alumina (13 wt % CuO on alumina) supplied by Sigma-Aldrich was crushed into a powder. This catalyst was used in the CO_2_ conversion tests and labeled CA.

#### Wet Impregnation

The CZAWI catalyst was prepared by wet impregnation of commercial CA with an aqueous solution of dihydrate zinc acetate (Sigma-Aldrich, 99%), similar to that described by Semelsberger et al., (2006) and Badmaev et al., (2015). In brief, 1 g of crushed CA was mixed with 16 ml zinc acetate 0.1 M and dried in a rotary evaporator (42 rpm) at 80°C for 1.5 h. Then, it was dried at 80°C in an oven overnight and calcined at 300°C for 2 h at 5°C/min. This procedure was carried out to obtain a Cu:Zn mass ratio of 1:1.

#### Coprecipitation

The coprecipitation method was used to prepare the catalyst labeled CZACP based on the methodology reported by Ereña et al., (2013) and Gayubo et al., (2014). The metal precursors were aqueous solutions of copper (II) nitrate trihydrate (Sigma-Aldrich), zinc acetate dihydrate (Sigma-Aldrich 99%), and aluminum nitrate nonahydrate (Sigma-Aldrich). 41.5 ml of Cu(NO_3_)_2_, 40.5 ml of Zn(CH_3_COO)_2_, and 33.3 ml of Al(NO_3_)_3_ aqueous solutions 0.1 M were mixed, and the corresponding metals were precipitated using a sodium carbonate (Sigma-Aldrich) solution as precipitating agent. Citric acid (Sigma-Aldrich) was used as a chelating agent. The precipitated solid was filtered, washed to remove alkaline cations, and dried at 70°C overnight. Then, the solid was calcined at 300°C for 2 h following a heating ramp of 5°C min^−1^.

### Catalyst Characterization

X-ray diffraction patterns were obtained in a Bruker D2 Phaser diffractometer operating at 30 kV and 10 mA with the Cu–Kα radiation in the 2θ range 15–70°.

Textural properties, such as specific surface area and pore and micropore volume, were obtained from nitrogen adsorption/desorption isotherms at −196°C using an Autosorb-iQ3 (Quantachrome Instruments, United States). Prior to the measurements, the catalysts were outgassed at 200°C for 6 h under a vacuum of 10^–6^ bar.

The chemical composition of the samples was evaluated by X-ray fluorescence (XRF) semi-quantitative analysis using an ARL ADVANT`XP + X-ray spectrometer (Thermo Scientific, United States).

### Adsorption Isotherms

Carbon dioxide and hydrogen adsorption isotherms were measured with the aid of a magnetic suspension balance (Rubotherm, Germany) at 50, 100, 150, and 200°C.

### Catalytic Tests

**FIGURE 1:**
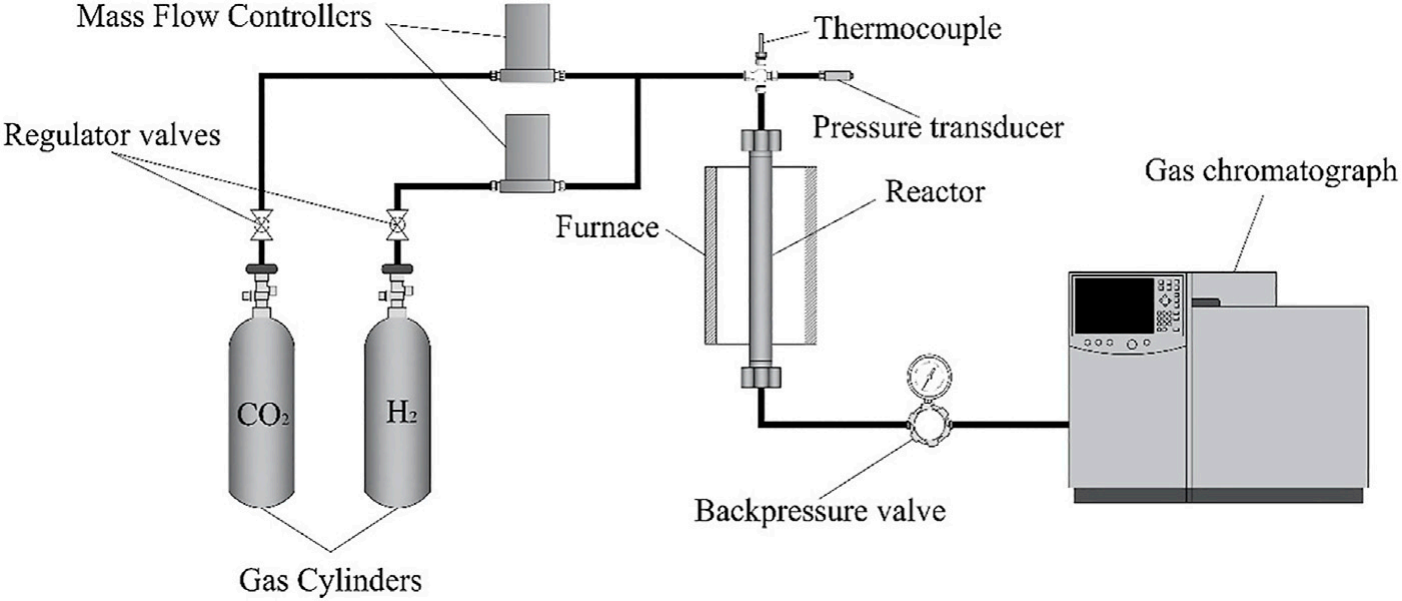
Experimental setup.

The catalytic reaction was carried out in a fixed-bed flow reactor, as shown in Figure 1. The reactor consisted of a column of 19 mm diameter and 300 mm long. The catalyst was placed in the central part of the column (about 30 mm long), and the catalyst bed was kept stationary by adding quartz wool on both sides. Prior to each run, the catalyst was reduced *in situ* under an H_2_ flow (15 ml min^−1^, 1 bar, 230°C, 2 h), aiming to reduce CuO species to Cu^0^. The system was then pressurized to 25 bar with H_2_, and after thermal equilibrium was reached, the reaction was carried out with a velocity of 1,200 ml g^−1^ h^−1^ (60 ml min^−1^, 1CO_2_:3H_2_ molar ratio) for 30 h. The reactor pressure was maintained at 25 bar by using a back-pressure-regulator (BPR) valve (Swagelok), the temperature was controlled by an electric resistance heater, and the flow rate of the reactant gas mixture was controlled by a Brooks^®^ mass flow controller. Products were analyzed by collecting samples at the exit of the column with the aid of a gas-tight sample valve, which were then injected into a gas chromatograph Agilent 7820 equipped with a flame ionization detector (FID) and a thermal conductivity detector (TCD).

CO_2_ conversion $\left(X_{CO_{2}}\right)$ and the product (methanol and DME) selectivity (S) and yield (Y) were defined as follows (Eqs 1–3):

$$X_{CO_{2}}\left(\%\right)=\frac{n_{in}-n_{out}}{n_{in}}\cdot 100,$$ (1)

$$S\left(\%\right)=\frac{y_{i}\cdot n_{p}}{n_{in}-n_{out}}\cdot 100,$$ (2)

$$Y\left(\%\right)=\frac{y_{i}\cdot n_{p}}{\;n_{in}}\cdot 100,$$ (3)

where *n*_*in*_ is the amount (mol) of CO_2_ in the inlet stream, *n*_*out*_ is the amount (mol) of CO_2_ in the outlet stream, *n*_*p*_ is the amount (mol) of the product in the outlet stream, and *y*_*i*_ is the stoichiometric coefficient of product *i*.

## Results and Discussion

### Characterization of the Catalysts

**FIGURE 2:**
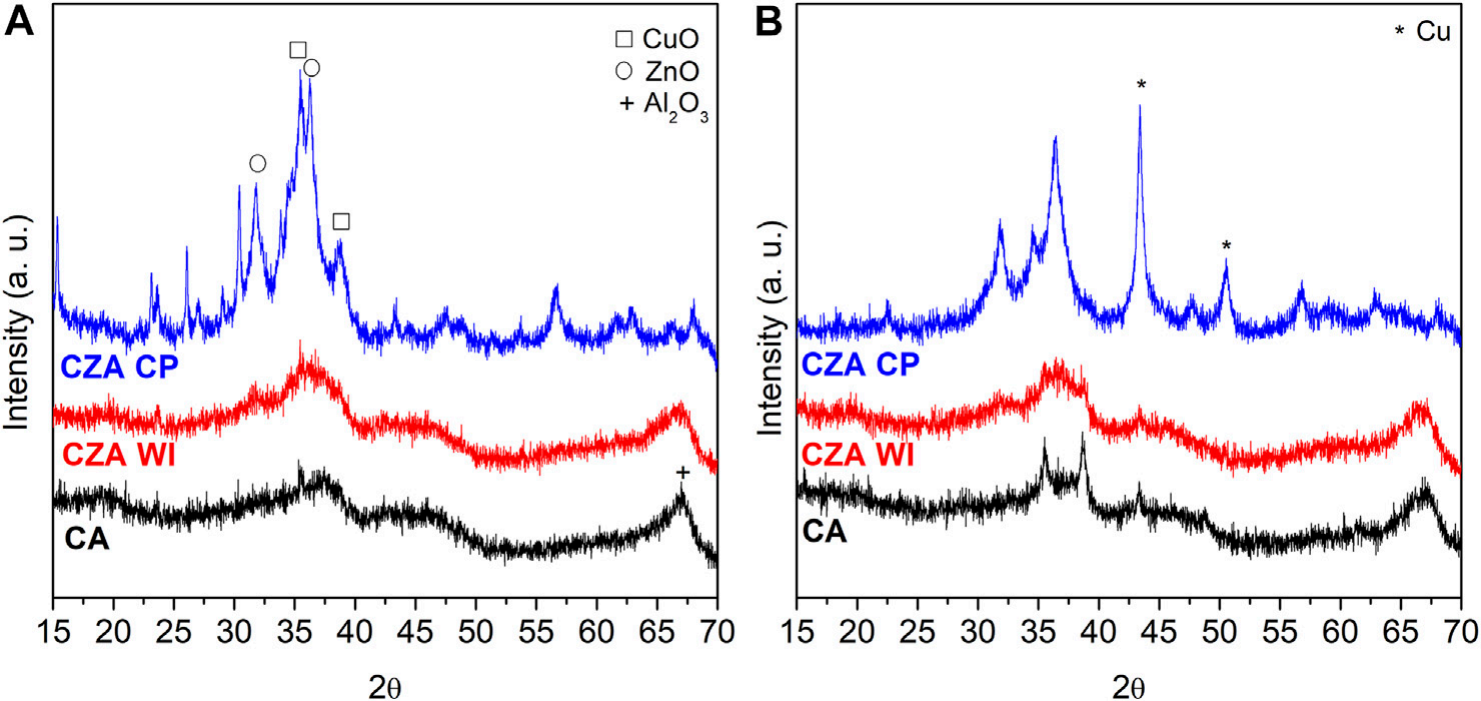
XRD patterns of the catalysts before **(A)** and after **(B)** reactional tests.

The XRD patterns for the commercial (CA), modified (CZAWI), and synthesized (CZACP) catalysts are shown in Figure 2. The impregnation of zinc does not significantly alter the amorphous structure of the commercial material. The patterns exhibit a peak at 2θ = 67°, indicating a poorly crystalline *γ*-Al_2_O_3_ mixed with the primary amorphous structures. A similar pattern is reported for a material prepared from a copper(II) nitrate aqueous solution to obtain catalysts supported by Al_2_O_3_ (Pires et al., 2015). The peaks corresponding to the CuO and ZnO phases cannot be observed probably due to high dispersion or low content in the catalysts. This behavior has already been observed in studies with metallic oxides supported on *γ*-Al_2_O_3_ (Afshar Taromi and Kaliaguine, 2018). Literature also reports that the diffraction peaks of CuO gradually decrease with increasing calcination temperature (Luo et al., 2005). After reaction in a fixed bed, only the spent CZACP sample presented a significant variation in the XRD pattern, suggesting structural modification, which can be related to the presence of residues of the precipitating agent, which is intrinsic to the preparation method.

**FIGURE 3:**
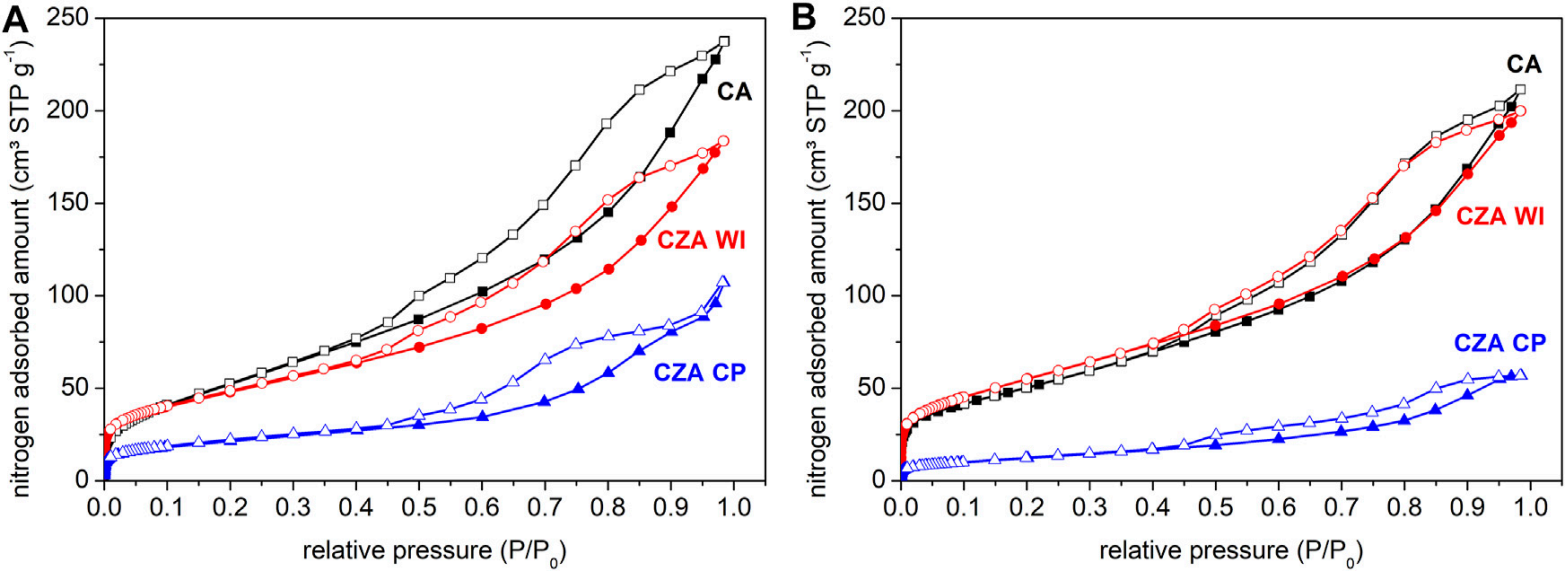
N_2_ adsorption isotherms at −196°C for catalysts **(A)** before and **(B)** after reaction.

Nitrogen adsorption isotherms are shown in Figure 3. The isotherms are type IV with hysteresis, which are typical features of mesoporous materials (Thommes et al., 2015). Table 1 summarizes the textural properties of the materials before and after the hydrogenation reaction, as calculated from the N_2_ adsorption/desorption isotherms. For the catalyst CZAWI, prepared by wet impregnation, both surface area and total pore volume decrease when compared to CA, which is very likely due to the loading of pores with zinc species upon impregnation. Similar behavior is also reported for supported catalysts by Badmaev et al., (2015) and Bonura et al., (2017). Interestingly, micropores seemed to be unaffected by the impregnation process. Sample CZACP presents the lowest surface area, similar to the values reported in the literature for CZA catalysts obtained by coprecipitation (Aguayo et al., 2007; An et al., 2007; Ereña et al., 2013). After the reaction, pore volume and specific surface area slightly decrease for CA and CZACP, whereas for CZAWI, a slight increase of these properties is observed toward comparable values as those of CA. Considering the sensitivity of the methods applied to determine those properties, it is fair to say that textural features remain practically unchanged, despite the observed loss of zinc that had been previously impregnated.

**TABLE 1:** Physical properties for samples.

Sample	Physical properties		
	$S_{BET}\left(m^{2}g^{-1}\right)$ ^a^	$V_{P}\left(cm^{3}g^{-1}\right)$ ^b^	$V_{MP}\left(cm^{3}g^{-1}\right)$ ^c^
Before reaction tests			
CA	205	0.34	0.06
CZAWI	168	0.26	0.06
CZACP	78	0.14	0.03
After reaction tests			
CA	186	0.30	0.07
CZAWI	192	0.29	0.07
CZACP	45	0.09	0.04

a Specific surface area.

b Total pore volume.

c Micropore volume.

The main elements detected by XRF in the commercial and prepared samples before and after the reaction tests are shown in Table 2 in terms of metal oxides. As expected, the commercial catalyst mainly comprises copper and aluminum oxides in the proportions reported by the supplier. The modified catalyst (CZAWI) presented zinc oxide, confirming that the wetness impregnation was effective in loading the metal into the sample. For CZACP, the same oxides with different composition were detected. After the reaction tests, the spent catalysts presented a slight decrease in CuO and ZnO at the expense of an increase in SiO_2_. For the spent CZACP sample, a relevant amount of Na_2_O has been detected, which consists of an unwashed residue of the precipitating agent, not found in the fresh sample. A possible cause for this discrepancy is sample heterogeneity with local variations in the composition of oxides. Nevertheless, even if Na_2_O was discounted from the composition basis, CuO and ZnO would still be decreasing after the reaction tests.

**TABLE 2:** Composition of samples in oxides by X-ray fluorescence.

Compound (% wt)	Samples		
	CA	CZAWI	CZACP
Before reaction tests			
Al_2_O_3_	81.52	68.79	12.22
CuO	16.89	15.57	27.20
ZnO	—	14.36	60.16
Si_2_O	0.40	0.37	0.18
Na_2_O	—	—	—
After reaction tests			
Al_2_O_3_	81.05	73.46	19.10
CuO	13.02	11.11	19.29
ZnO	—	9.33	43.32
Si_2_O	3.62	4.40	0.99
Na_2_O	—	—	13.54

### Adsorption Isotherms

**FIGURE 4:**
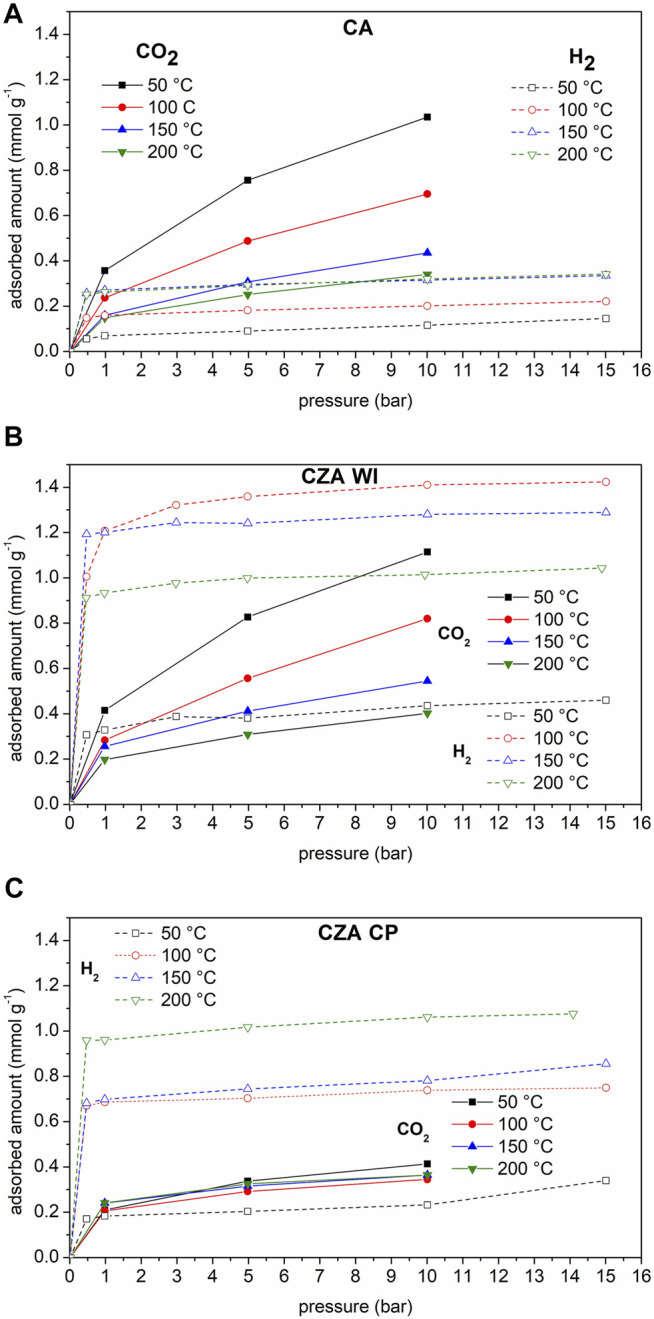
CO_2_ and H_2_ isotherms on **(A)** CA, **(B)** CZAWI, and **(C)** CZACP.

Figure 4 shows CO_2_ and H_2_ isotherms at different temperatures for (a) CA, (b) CZAWI, and (c) CZACP samples. All catalysts have a similar behavior regarding CO_2_ adsorption, that is, the uptake decreases with increasing temperature, thus indicating that it is predominantly a physical and exothermic phenomenon under the studied conditions. Adsorption capacity is similar for CA and CZAWI materials at all pressures and temperatures, although the material modified with zinc has a slight advantage, despite the reduction in textural properties. These results suggest that the presence of zinc improves the interaction of the material with CO_2_. Data of temperature programmed desorption of CO_2_ available in the literature (Gao et al., 2013) corroborate the increase in CO_2_ adsorption capacity for catalysts with ZnO. For the CZACP sample, the CO_2_ adsorption behavior is very distinct, with similar adsorption uptakes for the four temperatures under study.

Hydrogen isotherms, on the other hand, present a much more pronounced rise in uptake in the low-pressure range than CO_2_, indicating strong adsorbate–adsorbent interactions. One can observe that a temperature increase leads to higher H_2_ uptake for the commercial catalyst, whereas that modified with zinc (CZAWI) presents an optimum uptake with respect to the temperature but is always higher than that of CA for the studied temperature range. For CZACP, H_2_ uptake is comparable to that of CZAWI, but in this case, it increases monotonically with temperature.

**FIGURE 5:**
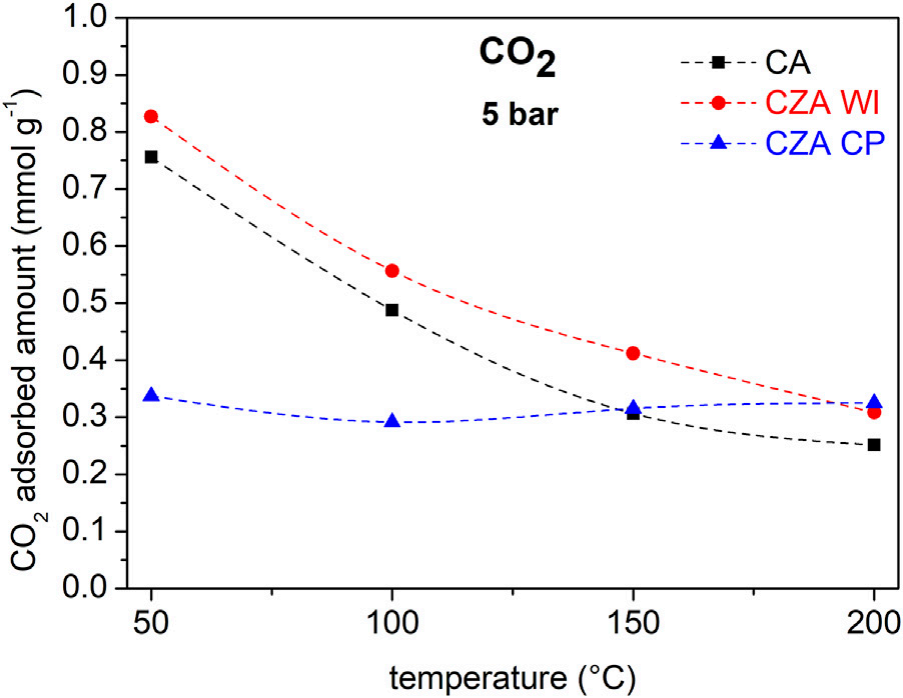
CO_2_ isobars on CA, CZAWI, and CZACP catalysts.

**FIGURE 6:**
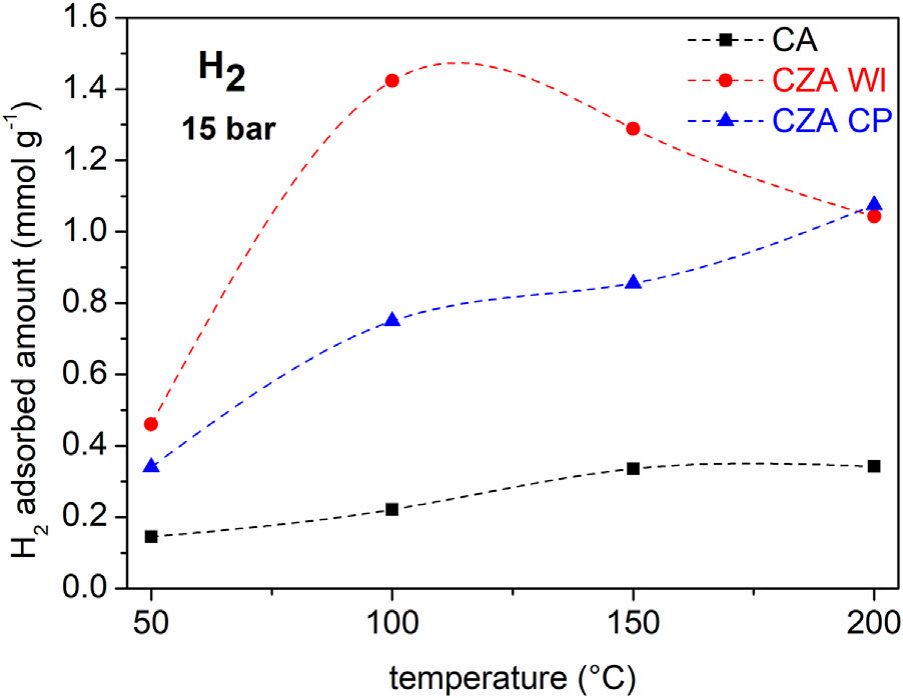
H_2_ isobars on CA, CZAWI, and CZACP.

To better evaluate the results with respect to temperature, the data are presented as isobars, as shown in Figures 5, 6 for CO_2_ and H_2_, respectively. The pressures of 5 bar for CO_2_ and 15 bar for H_2_ are representative of the reaction conditions used in the catalytic tests (total pressure 25 bar, H_2_:CO_2_ = 3:1). CO_2_ adsorption in sample CZAWI is superior to that of CA in this temperature range, whereas sample CZACP has much lower CO_2_ uptakes than CA and CZA at low temperatures. On the other hand, at 150°C it reaches a similar uptake to CA and at 200°C, the CO_2_ adsorption capacity is the highest. In Figure 6, it is notable that the samples CZAWI and CZACP have both higher H_2_ uptakes than the pristine commercial sample CA, which suggests that the H_2_ adsorption is influenced by the presence of zinc. The sample CZACP, despite the different zinc loading technique, has a similar H_2_ uptake as compared to sample CZAWI at 200°C, although the latter sample shows a maximum uptake close to 100°C, whereas the uptake in the former increases continuously with temperature.

Temperature programmed desorption measurements reported in the literature indicate that there is a direct relationship between CO_2_ and H_2_ adsorption capacity and the catalytic activity in methanol synthesis (Pokrovski and Bell, 2006; Xiao et al., 2015; Chang et al., 2017; Fang et al., 2019). This suggests that zinc-loaded catalysts should show a better performance than the commercial copper/alumina (CA) catalyst in the hydrogenation of CO_2_.

### Reaction Tests

**FIGURE 7:**
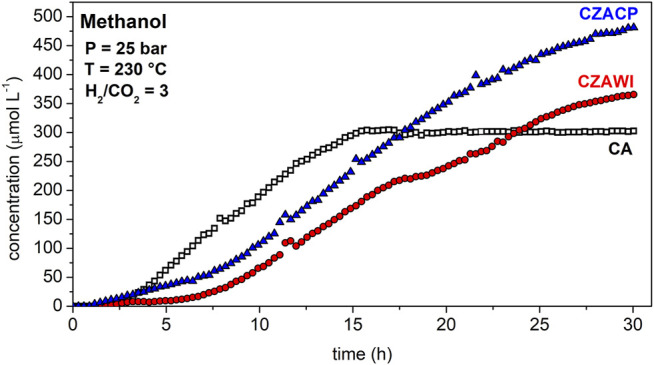
Methanol concentration at the outlet of the reactor using CA, CZAWI, and CZACP.

Figure 7 shows the methanol concentration at the reactor outlet using the two catalysts, CA and CZAWI. The commercial catalyst, CA, has a continuous methanol production for up to 15 h, when the concentration reaches a constant value close to 300 μmol L^−1^. This concentration is no longer modified until 30 h of reaction, indicating that the material does not lose activity during this time. For the CZAWI catalyst, methanol takes longer to be detected as a product, around 5 h of reaction, and its concentration increases more slowly when compared to the CA catalyst. The methanol production does not reach a steady value after 30 h of reaction. The CZACP catalyst has a similar methanol profile to that of CZAWI but with a higher concentration from the very first hours. According to the XRF results, this sample has the highest copper content, which may be associated with the presence of more available active sites for CO_2_ hydrogenation.

**FIGURE 8:**
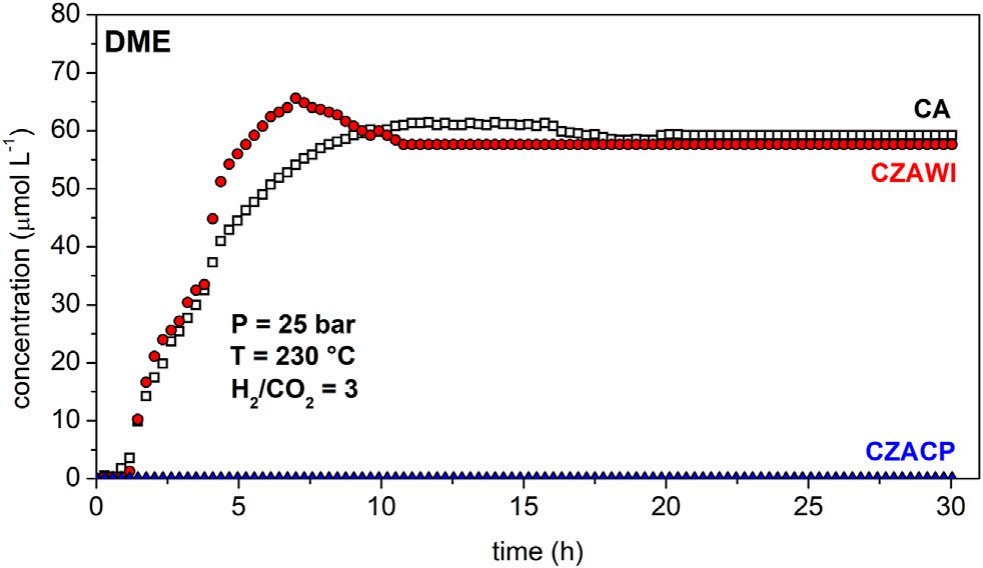
DME concentration at the end of the reactor using CA, CZAWI, and CZACP.

In addition to methanol, DME, CO, and water are also detected as reaction products. Figure 8 shows DME concentration history at the outlet of the reactor for the evaluated catalyst. CA and CZAWI catalysts showed a similar behavior regarding DME concentration, whereas no such product was observed for the sample CZACP. Unlike the methanol concentration history, after 10 h, the DME concentration reaches a constant concentration of about 60 μmol L^−1^ for both CA and CZAWI samples. DME is produced by methanol dehydration, which requires acidic sites in the catalyst in order to occur under the current conditions of pressure and temperature. The acid sites in the studied catalysts are provided by *γ*-Al_2_O_3_, which is present in the lowest concentration in the CZACP sample, according to the XRF results. This may be the reason for the negligible production of DME when using this catalyst.

**FIGURE 9:**
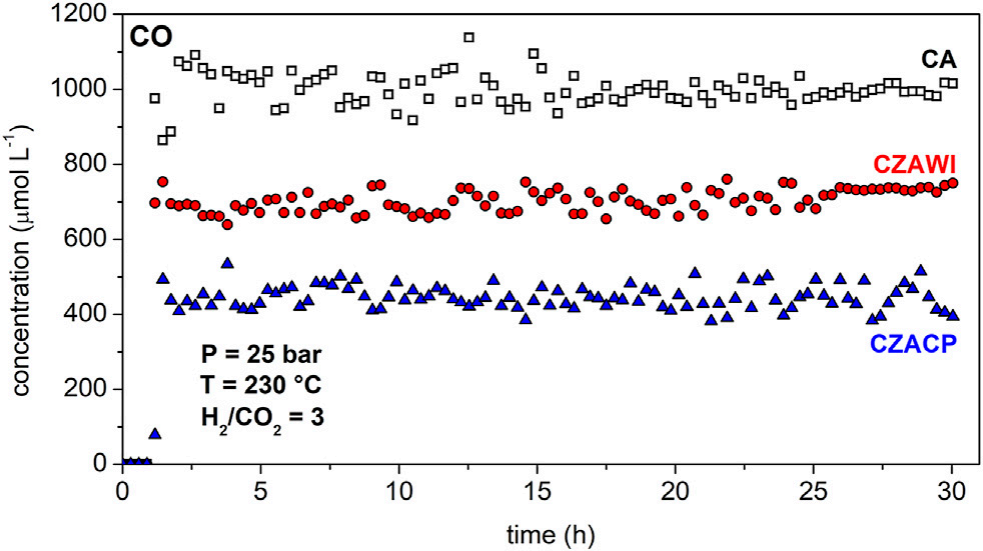
CO concentration at the end of the reactor using CA, CZAWI, and CZACP.

Figure 9 shows the CO concentration history at the reactor outlet for each catalyst. The concentration of carbon monoxide follows the same behavior in all cases: after 5 h of reaction, the concentration reaches a plateau that remains for the entire period that the reaction was monitored. However, this plateau has different values for each catalyst. For the commercial material, CA, the CO concentration reaches approximately 1,000 μmol L^−1^; with the CZAWI catalyst, a concentration of 750 μmol L^−1^ of CO was obtained; and for the CZACP, the concentration of CO was about 450 μmol L^−1^. The lower CO concentration is probably related to methanol formation, which was higher for CZACP.

The concentration histories of the reaction products show that regardless of the catalyst sample, methanol production is much slower than DME and CO formation. This indicates that methanol dehydration to form DME and the reverse water–gas shift (RWGS) reaction producing carbon monoxide have faster kinetics than the CO_2_ hydrogenation reaction, which is, therefore, the rate-limiting step of the global reaction rate (Aguayo et al., 2007; Qin et al., 2015). Although methanol is rapidly dehydrated to form DME and slowly produced from the carbon dioxide hydrogenation, it is still detected at the outlet of the reactor because methanol is also produced by the reaction of carbon monoxide hydrogenation (Alvarez et al., 2017; Battaglia et al., 2021).

**FIGURE 10:**
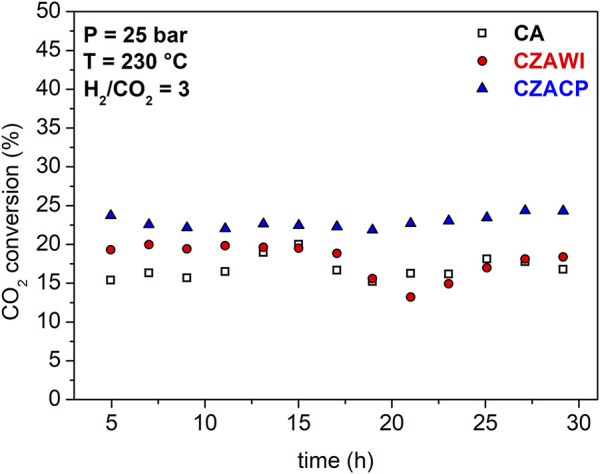
CO_2_ conversion with time using CA, CZAWI, and CZACP.

Figure 10 shows the transient CO_2_ conversion for the catalysts under study in this work. CO_2_ conversion reaches a constant value in the first hours of reaction for CZACP, unlike the CA and CZAWI catalysts, which present variation with respect to a trend. However, after 24 h of reaction, no significant variations are observed in the conversion for both materials.

**FIGURE 11:**
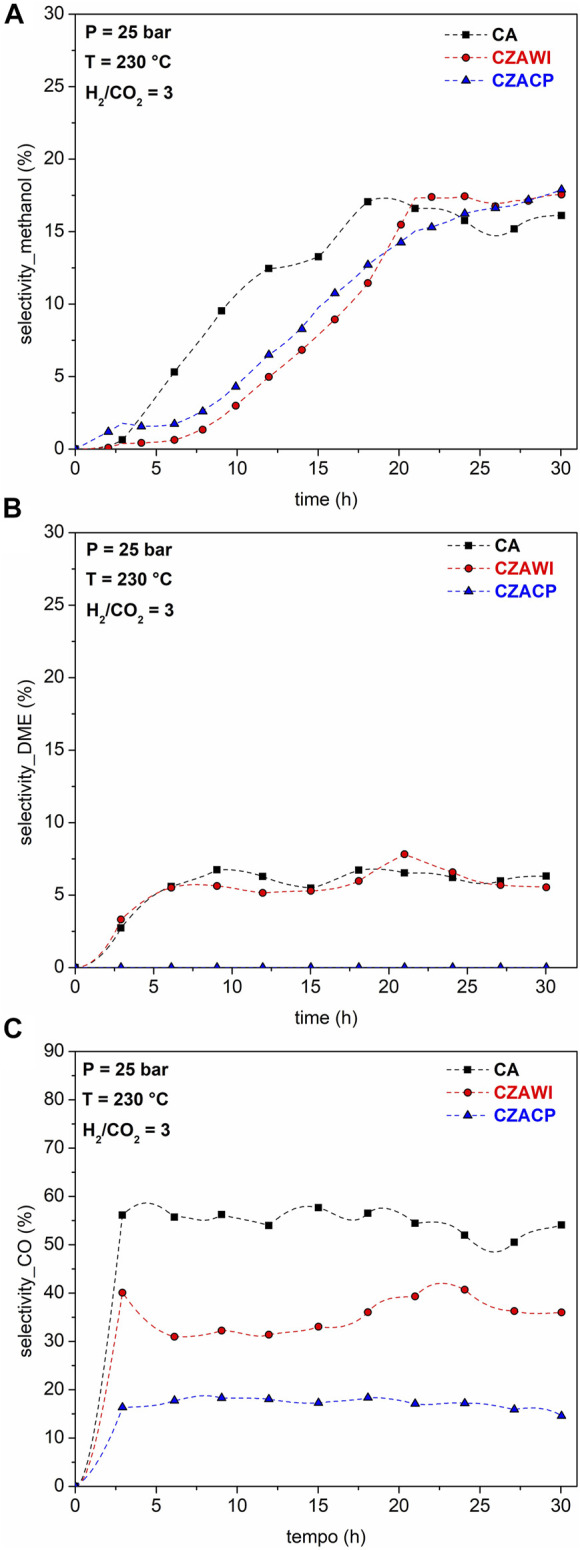
Selectivity of methanol **(A)**, DME **(B)**, and carbon monoxide **(C)** for tested catalysts.

The selectivity of products is shown in Figure 11. Methanol selectivity increases until a constant value is reached after 20 h of reaction for both CA and CZAWI catalysts, while for CZACP, there is a rising trend at 30 h of reaction. For DME, the selectivity reaches steady values for CA and CZA after 5 h of reaction. The carbon monoxide selectivity profiles quickly reach a steady state, at which CA and CZACP samples show the highest and lowest CO selectivity, respectively.

**FIGURE 12:**
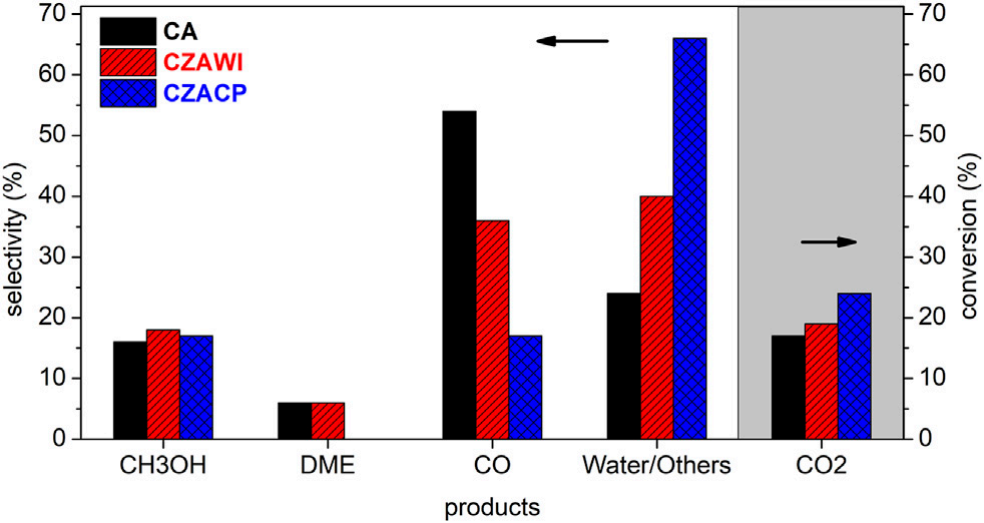
Product selectivity and CO_2_ conversion (hatched area) at a steady state.

**FIGURE 13:**
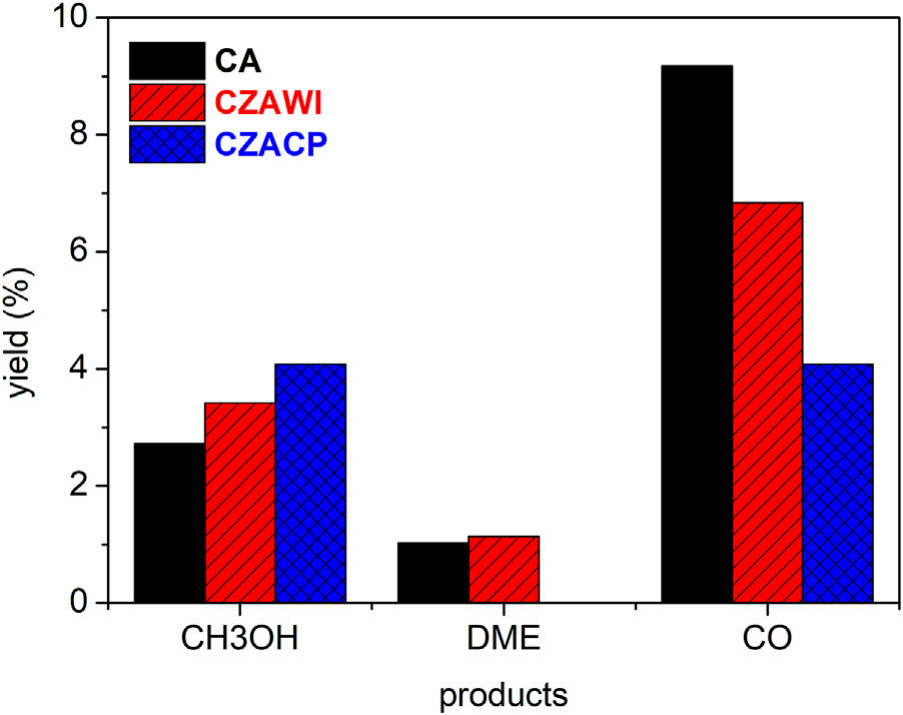
Product yield at a steady state.

Figure 12 summarizes the CO_2_ conversion and selectivity calculated according to Eqs 1, 2, respectively, for the products identified in the CO_2_ hydrogenation reaction after 30 h when a steady state was roughly reached. Figure 13 shows the product yield, estimated according to Eq. 3, under the same conditions.

The most active catalyst in this study was the one produced by coprecipitation (CZACP), presenting a CO_2_ conversion of 24%. The highest yield for methanol and the lowest yield for CO were obtained with this catalyst. The catalytic performance obtained in this study is comparable to that reported in the literature for the direct synthesis of DME (Aguayo et al., 2007; Ateka et al., 2017; Ateka et al., 2018).

By correlating the catalytic activity with the adsorption isotherms of the reactants, it was possible to confirm that the catalyst with the highest adsorption capacity for CO_2_ and mainly H_2_, CZACP, is the one that shows a better performance in the hydrogenation of CO_2_ to form methanol. Hence, there is strong evidence that the reaction mechanism is based on the adsorption of hydrogen onto the material, in agreement with the mechanism proposed by Graaf et al. (1988).

## Conclusion

The commercial material CA showed activity comparable to the results obtained in the literature for the methanol production under the studied conditions. DME was produced more likely due to the acid sites introduced by *γ*-Al_2_O_3_ on this catalyst.

Even though zinc loading decreases the textural properties of the CA catalyst, it increases the interaction with CO_2_ and H_2_, thus improving the adsorption capacity when compared with the pristine catalyst. CO_2_ conversion, methanol selectivity, and yield were improved by the presence of ZnO, while DME production was not affected. Furthermore, DME formation was suppressed in the sample that was loaded with zinc by coprecipitation.

Although CA and CZAWI presented results that indicate potential application in methanol production, the CZACP material showed better CO_2_ conversion and higher methanol yield, in addition to producing less CO. This suggests that it enhances the CO_2_ hydrogenation reaction rather than the RWGS reaction.

In summary, the main impact of this work is regarding the insights on the reaction mechanism provided by the CO_2_ and H_2_ adsorption isotherms on the catalysts. The adsorption capacity of the reactants, evaluated under temperature and pressure conditions close to the hydrogenation reaction conditions, correlates well with the catalytic activity and hence the conversion and yield. This novel approach can be used to evaluate and aid the development of new catalysts.

## Data Availability

The original contributions presented in the study are included in the article/Supplementary Material; further inquiries can be directed to the corresponding author.
